# Modifiable risk factors of congenital malformations in bale zone hospitals, Southeast Ethiopia: an unmatched case-control study

**DOI:** 10.1186/s12884-020-2827-0

**Published:** 2020-02-27

**Authors:** Alemayehu Gonie Mekonnen, Alemu Girma Hordofa, Tamiru Tesfaye Kitila, Adem Sav

**Affiliations:** 1 Department of Nursing, College of Health Sciences, Debre Berhan University, Po. Box, 445 Debre Berhan, Ethiopia; 2 Department of Surgery, Goba Referral Hospital, Maddawalabu University, Bale Robe, Ethiopia; 3 Department of Nursing, College of Health Sciences, Ambo University, Ambo, Ethiopia; 4 School of Public Health and Social Work, Faculty of Health, Queensland University of Technology, Brisbane, Queensland Australia

**Keywords:** Birth defects, Congenital malformation, Infants, Ethiopia

## Abstract

**Background:**

Congenital malformations are structural, functional, and metabolic defects that develop during the organogenesis period and present at birth or later in life. There has been little research on congenital malformations in Ethiopia, knowledge on the incidence of birth defects at birth is unknown and the etiologies of the anomalies are limited. This study, therefore, aimed to assess the modifiable risks of congenital anomalies among women in Bale zone hospitals, Ethiopia.

**Methods:**

An unmatched case-control study was conducted from February 2018 to January 2019 in the Bale zone; namely Goba referral hospital, Robe, Ginnir and Dolomena hospitals. A total of 409 women were selected. Mothers who gave birth with any type of congenital malformation were assigned as cases and those who gave live births without any congenital abnormalities were assigned as controls. Controls were selected by the lottery method from the labor ward. For each case, two consecutive controls were included. Data were entered into Epi-data 3.1 and exported into Statistical Package for the Social Sciences (SPSS) version 21 for analysis. Logistic regression was conducted to analyze the data.

**Results:**

Alarmingly, women who had been exposed to pesticides during the current pregnancy were two times more prone to give congenital malformed infants than their counterparts (AOR = 3.19; 95% CI = 1.31, 10.96). Additionally, those women who chewed *khat* during the periconceptional period were two times more likely to have congenital malformed infants as compared to women who did not engage in this activity (AOR = 2.40; 95% CI = 1.11, 5.19).

**Conclusions:**

Urgent attention needs to be given by public health professionals and services to *khat* chewing and maternal exposure to pesticides during pregnancy to reduce the risk of congenital malformations.

## Background

Congenital malformations (CM) are structural, functional, and metabolic defects that develop during the organogenesis period and present at birth or detected later in life [1]. These malformations are present in 3% of all newborns and responsible for nearly 7% of neonatal deaths worldwide [2]. Congenital malformations can be caused by genetic, chromosomal, environmental, multifactorial effects, and micronutrient deficiencies or unknown etiological agents [3, 4]. A strong association has been reported between maternal pre-pregnancy obesity and certain birth defects [5, 6]. Although genetic factors account for 20% of the risks, the majority of birth-defects are environmental factors [1, 3]. For example, environmental teratogens (exposure to medicines, certain environmental chemicals, alcohol intake) have been reported to have an adverse and disruptive effect on the embryo or the fetus [7–9]. Environmental teratogen has mechanical effects on the vascular and the amnion disruptions by increasing the risk of malformation pathology [6, 10]. Maternal infections with syphilis and rubella are also stated as the risk of birth-defects in low and middle-income countries [11].

The incidence of congenital malformation varies widely from country to country [2] and from region to region, with a range of 1.73 to 6.3% [12–14]. For example, it was reported to be 1.7% in Brazil [15], 2.22% in Eastern India [16], 3.63% in Iraq [17], 6.3% in Nigeria 6.3% [14]. One such country with high rates of congenital malformation is Ethiopia. In central and north-west Ethiopia, the proportion of congenital anomaly was 199 per 10,000 children who visited the hospitals [18]. This is not surprising as evidence indicated that the highest numbers of birth defects occurred in low and middle-income countries where environmental teratogenic risk factors were more problematic as compared to high-income countries [2, 19]. Indeed, there was evidence that suggested childbearing-age women in developing countries like Ethiopia are exposed to potential teratogenic risks like infectious agents and environmental chemical compounds [20, 21]. For example, in a study conducted in Addis Ababa showed that women were exposed to high concentrations of metals, nitrates, coliform, and other pathogens which might cause birth-defects [22].

There was widespread recognition that the prevention of congenital malformations during pregnancy requires risk identification [11]. Indeed, the identification of modifiable risk factors of birth defects provide an opportunity for primary prevention which include prevention of sexually transmitted infections, promoting healthy dietary habits and fortification of foods with folic acid, and preventing maternal infections during the periconceptional period (1 month before conception and 1 month after conception) [23]. However, in order to identify, such modifiable risk factors that have been associated with a congenital malformation, health care providers need evidence-based information. More specifically, they need to know which modifiable risk factors can lead to congenital malformation.

Knowledge about congenital anomalies is useful to identify clues to risk factors of conditions, for health services planning and evaluating antenatal screening in populations with high risk [2, 17]. Based on this premise, many studies have investigated the risk factors of congenital anomalies [21, 24, 25]. Although these studies have increased our awareness of the link between modifiable risk factors and congenital malformations, some have significant limitations such as study design, lack of proper sample size, and lack of control group [11, 16]. In Ethiopia, there is a scarcity of research on congenital malformations. There is currently very limited knowledge on the incidence of CMs at birth and the etiologies of the anomalies are limited. Besides, the incidence of CM and the modifiable risk factors could be significantly different in Ethiopia than high-income countries (where most research has occurred), because of unique cultural and societal issues in this country. Without such knowledge, healthcare professionals will have very limited ability to identify Ethiopian women who are at risk of developing CMs and they have been limited in implementing effective treatment programs. Hence, the first step in preventing and treating the incidence of CMs at birth in Ethiopia could be understanding the modifiable risk factors that are likely to lead to this health issue. This study, therefore, aimed to assess the modifiable risks of congenital malformations in Bale zone hospitals, Ethiopia, a country where there are high rates of CMs.

## Methods

### Study area and period

The study was conducted from February 2018 to January 2019 in Bale zone hospitals; namely Goba referral hospital, Robe, Ginnir and Dello-Menna hospitals in Ethiopia. The Bale zone is located in the southeastern part of Ethiopia. Robe, the zone city, is located 435 km far from the capital city of Ethiopia; Addis Ababa. Based on the 2007 census conducted by the Central Statistical Agency, this Zone has a total population of 1,402,492 of which 26.20% are urban inhabitants and 3.18% are pastoralists.

The above-mentioned hospitals provide almost all types of obstetric care and babies are routinely screened for congenital abnormalities before discharge from the maternity unit. In the study hospitals, the exact number of birth defects was unknown, but on average, 6 congenital anomalies have been screened in each hospital per month (Bale zone health office report 2018).

#### Study design

An unmatched case-control study design was employed to achieve the objectives.

#### Study population

All infants born from February 2018 to January 2019 were the total study population. Mothers who gave birth with any type of congenital malformation were assigned as cases and mothers who gave live birth without birth defects were assigned as controls. Minor defects were excluded from the study because of the difficulties in ascertaining such defects. If multiple CMs were diagnosed, the primary major birth-defect was taken (spinal bifida with clubfoot, spinal bifida was taken).

### Sample size determination

The sample size was calculated using Fleiss formula from the Open-Epi software package with considering the following unmatched case-control study parameters (proportion difference approach) [26]: The proportion of the desired confidence level = 95%. The power (chance of detecting) = 80%. The least extreme odds ratio to be detected (the strength of relationship expected to be found among cases & controls) =2. The ratio of the case to control was 1:2. Hypothetical percentage of exposure among controls by considering the most common congenital malformations (cleft lip) =18% [3]. Using these values, the percent of expected exposure among cases was 30.5%. With the above assumptions and by adding a non-response rate of 5%, the final sample size was 423 (141 cases and 282 controls).

### Sampling procedure

All mothers who delivered infants with congenital anomalies within the study period were invited to participate in the study. Congenital malformation was diagnosed by Obstetricians at the time of delivery. Mothers who had delivered infants with no congenital anomalies were also recruited as a control group. Controls were selected from the same ward as the same day of case incidence. The required numbers of controls were selected by the lottery method from the labor ward. For each case, two consecutive controls were included.

### Data collection

A structured and pre-tested questionnaire was employed to collect data (Additional file 1). The questionnaire was prepared first in English from published articles and then translated into Amharic and Afan Oromo (local languages) by expertise, and its reliability and validity were confirmed with 5% of the sample. Data collectors and supervisors were trained by principal investigators before the data collection. Data on the following variables were collected in the questionnaire: socio-demographic characteristics, obstetric characteristics (birth interval, number of children, antenatal care follow up, use of contraceptives), maternal medical histories and toxic or environment exposure status.

### Operational definition

#### Cases

Are those mothers who gave birth with any type of congenital malformation. **Controls:** are those mothers who gave live birth without any identified congenital malformation.

#### Congenital malformations

are defined as structural, functional, and metabolic defects that develop during the organogenesis period and detected at birth. The diagnosis of congenital anomalies was confirmed through clinical examination during childbirth. In this study, the terms; birth defects, congenital abnormalities, and congenital malformations are synonymous, and we used the terms interchangeably throughout the document.

### Data processing and analysis

Data were checked for completeness and inconsistencies. Epi-data version 3.1 was used for data entry and data were exported into Statistical Package for the Social Sciences (SPSS) version 21. Descriptive statistics were computed. To identify the factors, which were significantly associated with the risk of congenital malformation, multiple logistic regression was performed. Independent variables that had a significant association in the bivariate analysis were entered into the multivariable analysis. In the final model, a significant association was declared at a *p < 0.05*. The results were presented in text and tables with adjusted odds ratio (AOR) and the corresponding 95% confidence interval.

### Ethical consideration

Ethical approval was obtained from a research review committee of Madawalabu University. Letters were secured from the Bale zone Health Bureau and respective hospitals. Written informed consent was obtained from each study participant. All information obtained from each study participant was kept confidential throughout the study, and the name of the participant was replaced by code. Withdrawal from the study at any point if they wished was assured.

## Results

### Maternal socio-demographic characteristics

A total of four hundred nine women (136 cases and 273 controls) were successfully interviewed via questionnaires, giving a response rate of 96.7% for both cases and controls. About 35 % (34.8%) of controls and nearly one-third of cases (29.4) were in the age group of 21–25 years. The largest proportions of respondents, 96.3% of cases and 97.1% of controls were married. Around 64 % of women among cases and 60.8% of women among controls were Muslim. The majority of women (65.4% of cases versus 55.0% of controls) were housewives. Concerning the educational level of the respondents, 37.5% of cases and 27.8% of controls did not attend formal education and a small proportion of participants completed college education (13.9% of cases versus 20.5% of controls). Almost half (52.2%) of cases lived in an urban area (Table 1).

**Table 1:** Sociodemographic characteristics of mothers in Bale zone hospitals, Ethiopia, January, 2019

Characteristics	Categories	Cases n (%)	Controls n (%)
Age of respondents (in years)	≤20	16 (11.8)	31 (11.3)
	21–25	40 (29.4)	95 (34.8)
	26–30	37 (27.2)	83 (30.4)
	31–35	25 (18.4)	41 (15.1)
	≥36	18 (13.2)	23 (8.4)
Marital status of respondents	Married	131 (96.3)	265 (97.1)
	Single/separated	5 (3.7)	8 (2.9)
Religion of respondents	Muslim	87 (64.0)	166 (60.8)
	Orthodox	42 (30.9)	83 (30.4)
	Protestant	5 (3.7)	17 (6.2)
	Catholic	2 (1.4)	7 (1.6)
The educational level of respondents	Illiterate	51 (37.5)	76 (27.8)
	Primary education	43 (31.6)	68 (24.9)
	Secondary education	23 (17.0)	73 (26.8)
	College education	19 (13.9)	56 (20.5)
Occupation of respondents	Housewife	89 (65.4)	145 (55.0)
	Employed	18 (13.2)	45 (16.5)
	Farmer	15 (11.1)	41 (15.1)
	Marchant	11 (8.1)	38 (13.9)
	Others (daily laborer & student)	3 (2.2)	4 (1.5)
Residence of respondents	Urban	71 (52.2)	167 (61.2)
	Rural	65 (47.8)	106 (38.8

### Obstetrics characteristics of respondents

Out of the total study participants, nearly half of both controls (48.6%) and cases (44.1%) were multigravidas. Twenty-one percent (21.6%) of cases and 18.4% of controls reported a history of abortion/pregnancy loss before 28 weeks of gestation. A higher percentage of cases (4.1%) and 2.3% of controls reported that they had a family history of anomalies. Around 42 % of cases and a half (49.6%) of controls utilized contraceptives before the current pregnancy (Table 2).

**Table 2:** Obstetrics characteristics of mothers in Bale zone hospitals, Ethiopia, January 2019

Obstetrics Characteristics	Categories	Cases	Controls
Gravida	Primigravida	39 (28.7)	66 (24.2)
	Multigravida	61 (44.8)	132 (48.3)
	Grand multigravida	36 (26.5)	75 (27.5)
Gestational age	< 28 weeks	19 (14.0)	0
	≥28–37 weeks	70 (51.5)	12 (4.4)
	≥37 weeks	47 (34.5)	261 (95.6)
Number of infants delivered	Single	111 (81.6)	262 (95.9)
	Twin and above	25 (18.4)	11 (4.1)
Fetal sex	Female	65 (48.4)	141 (51.6)
	Male	64 (47.1)	132 (48.4)
	Undefined	7 (5.5)	0
Did you have previous pregnancy neonatal death? (*n* = 304)	Yes	5 (5.2)	6 (2.9)
	No	92 (94.8)	201 (97.1)
Did you have an abortion history/pregnancy loss before 28 weeks? (n = 304)	Yes	20 (21.6)	37 (18.4)
	No	77 (79.4)	169 (81.6)
Did you have a family history of anomalies? (n = 304)	Yes	4 (4.1)	6 (2.3)
	No	93 (95.9)	201 (97.7)
Did you use contraceptives before the current pregnancy?	Yes	40 (41.3)	102 (49.6)
	No	57 (58.7)	105 (50.4)

### Maternal medical histories

There was no major difference between maternal previous medical histories (pregnancy-induced hypertension, diabetes, and hyperthyroid disorder) and having congenital malformed infants. Nearly equal proportion of respondents, 6.6% of cases and 7.3% of controls reported a history of pregnancy-induced hypertension. A few percent (2.2%) of cases had been diagnosed with the hyperthyroid disorder before the current birth. Similarly, less than 2 % of the cases and 2.2% of controls reported a history of diabetes before the current birth. Regarding the prior family history of anomalies, 7.4% of the cases and 2.5% of controls reported a family history of anomalies. About 12.9% of cases and 9.1% of controls have been diagnosed with anemia during current pregnancy (Table 3).

**Table 3:** Medical histories of mothers in Bale zone hospitals, Ethiopia, January 2019

Maternal medical histories	Categories	Cases n (%)	Controls n (%)
Did you have a history of pregnancy-induced hypertension?	Yes	9 (6.6)	20 (7.3)
	No	108 (79.4)	221 (81.0)
	Do not know	19 (14.0)	32 (11.7)
Did you have a family history of anomalies?	Yes	10 (7.4)	7 (2.5)
	No	104 (76.5)	227 (83.2)
	Do not know	22 (16.1)	39 (14.3)
Did you have been diagnosed with a hyperthyroid disorder?	Yes	3 (2.2%)	8 (2.9)
	No	122 (89.7)	237 (86.8)
	Do not know	11 (8.1)	28 (10.3)
Did you have a history of diabetes?	Yes	2 (1.5)	6 (2.2)
	No	98 (72.0)	211 (77.3)
	Do not know	36 (26.5)	56 (20.5)
Did you have been diagnosed with anemia?	Yes	17 (12.5)	24 (8.8)
	No	98 (72.0)	210 (76.9)
	Do not know	21 (15.5)	39 (14.3)

### Toxic or environment exposure status

As can be seen from Table 4, a slightly higher proportion of women who had congenital malformed infants had been exposed to toxic environments. Thirteen percent (13.2%) of cases and 4.2% control mothers have been exposed to pesticides during pregnancy. Twenty-one percent (21.1%) of cases and 8.8% of control mothers reported that they were chewing *khat*^1^ during the periconceptional period (1 month before conception and 1 month after conception). The reported consumption of alcohol during the current pregnancy was 11.2 and 4.8% for case and control women, respectively. Around half (49.3%) of cases and 61.5% of control mothers reported that they had a separate cooking kitchen.

**Table 4:** Environment exposure status of mothers in Bale zone hospitals, Ethiopia, January 2019

Toxic or environment exposure status	Category	Cases N (%)	Controls N (%)
Have you been exposed to *pesticides* during pregnancy?	Yes	18 (13.2)	11 (4.1)
	No	118 (86.8)	262 (95.9)
Did you smoke a *cigarette* during the current pregnancy?	Yes	4 (2.9)	4 (1.5)
	No	132 (97.1)	269 (98.5)
Did you ever chew *khat* during the peri-conceptional period?	Yes	28 (21.1)	24 (8.8)
	No	108 (79.4)	249 (91.2)
Have you consumed/*drank alcohol* during the current pregnancy?	Yes	16 (11.2)	13 (4.8)
	No	120 (88.8)	260 (95.2)
Have you been exposed to *organic solvents (chemicals used in industries or installations* e.g. *benzene, methanol)* during the pregnancy?	Yes	6 (4.4)	6 (2.2)
	No	130 (95.6)	267 (97.8)
Have you been exposed to *heavy metals (mercury, lead, arsenic, aluminum)* during the current pregnancy?	Yes	3 (2.2)	5 (1.9)
	No	133 (97.8)	268 (98.1)
Have you been undergone *X-ray/radiation therapy* during pregnancy?	Yes	2 (1.4)	3 (1.1)
	No	134 (97.6)	270 (98.9)
Did you use a separate cooking kitchen?	Yes	65 (47.8)	153 (56.0)
	No	71 (52.2)	120 (44.0)
Was there any ventilation during heating/cooking?	Yes	67 (49.3)	168 (61.5)
	No	69 (50.9)	105 (38.5)
Have you used a *coal stove* for heating?	Yes	98 (72.1	213 (78.0)
	No	38 (27.9	60 (22.0)

### The type of congenital malformation

**Fig. 1:**
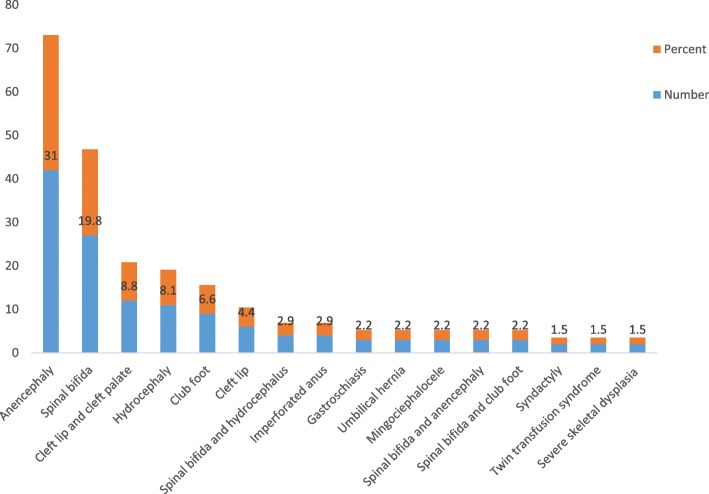
The type of congenital malformation in Bale zone hospitals, Ethiopia, January 2019

As shown in Fig. 1, various types of congenital malformations were diagnosed during the study period. Out of 136 congenital malformations, 42(31.0%) of them were anencephaly and 27(19.8%) were spinal bifida. Of the anomalies, the proportion of gastroschaisis, umbilical hernia, and meningoencephalocele were 2.2% (Fig. 1).

### Risk factors associated with congenital malformation

The bivariate logistic regression analyses were performed between independent variables and congenital malformation. The analyses revealed that women’s occupation, residence, number of infants, have been exposed to pesticides during pregnancy, have been drinking alcohol during pregnancy, and have been chewing *Khat* during the periconceptional period were statistically significant with a congenital malformation in the bivariate model. Those variables which had a significant association with a congenital malformation in the bivariate model were entered into multiple logistic regression analyses. The results of the analysis showed that women who have been exposed to pesticides during the current pregnancy were three times more prone to give congenital malformed infants than their counterparts (AOR = 3.19; 95% CI = 1.31, 10.96). Likewise, the likelihood of giving congenital malformed infants was significantly higher among women who chewed *khat* during the periconceptional period. In fact, women who chewed *khat* during their periconceptional period were two times more likely to have congenital malformed infants as compared to women who did not engage in this activity (AOR = 2.40; 95% CI = 1.11, 5.19) (Table 5).

**Table 5:** The analysis of risk factors associated with a congenital malformation in Bale zone hospitals, Ethiopia, January 2019

Characteristics	Category	Case	Control	COR (95% CI)	*p*-value	AOR (95% CI)	p-value
Occupation of women	Housewife	89 (65.4)	145 (55.0)	2.12 (1.76, 7.31)	0.031	2.79 (0.28,4.47)	0.171
	Employed	18 (13.2)	45 (16.5)	1.38 (0.49, 8.32)	0.210	1.03 (0.91,7.90)	0.125
	Farmer	15 (11.1)	41 (15.1)	1.26 (0.44, 3.01)	0.545	1.55 (0.88,15.54)	0.725
	Marchant	11 (8.1)	38 (13.9)	1		1	
Residence of women	Urban	71 (52.2)	167 (61.2)	0.69 (0.54, 1.81)	0.011	0.56 (0.43,5.83)	0.160
	Rural	65 (47.8)	106 (38.8	1		1	
Number of infants delivered	Single	111 (81.6)	262 (95.9)	0.19 (0.17,0.82)	0.035	0.23 (0.97,7.84)	0.243
	≥Twin	25 (18.4)	11 (4.1)	1			
Exposed to *pesticides* during pregnancy	Yes	18 (13.2)	11 (4.1)	3.63 (1.43,9.63)	0.001	3.19 (1.31–10.96)	0.001
	No	118 (86.8)	262 (95.9)	1		1	
D*rank alcohol* during the current pregnancy	Yes	16 (11.2)	13 (4.8)	2.67 (1.05,7.81)	0.041	2.59 (0.93,7.25)	0.070
	No	120 (88.8)	260 (95.2)	1		1	
*Khat* chewing during peri-conception	Yes	28 (21.1)	24 (8.8)	2.69 (3.51,8.72)	0.002	2.40 (1.11,5.19)	0.004
	No	108 (79.4)	249 (91.2)	1		1	

## Discussions

This is one of the few studies that shed light on the understanding of the modifiable risk factors of congenital malformations in Southeast Ethiopia, thereby helping health professionals and health services to tailor suitable treatment and prevention programs in this context. In an era of increasing pressures on health systems worldwide [27], our study brings us one step close to ensuring that by understanding the modifiable risk factors for CM, and how health services are delivered efficiently and optimally. In this study, maternal obstetric and medical histories did not have significant associations with delivering congenital malformed infants. This could be because obstetric and medical history variables did not affect the formation of congenital malformation. However, our result showed that women who have been exposed to pesticides during pregnancy and who chewed *khat* during their periconceptional period were at higher risk to deliver congenital malformed infants. Alarmingly, we found that women who had been exposed to pesticides during the current pregnancy were two times more prone to give congenital malformed infants than their counterparts. This adds to the overwhelming body of evidence on the negative impact of pesticide exposure on pregnancy outcomes worldwide. For example, a study conducted in Southern France revealed that being exposed to pesticides during pregnancy was associated with the risk of fetal congenital malformation [28]. Bianchi F et al. also reported that the association between birth defects and being exposed to textile dye [25]. In agreement with our results, the authors reported that being exposed to chemicals during pregnancy played a significant role in having an infant with a major birth defect [13, 29].

Our study adds to this body of evidence by suggesting that exposure to toxic chemicals during pregnancy has a detrimental impact on pregnant women in counties where there is very little or limited evidence (e.g., Ethiopia). With the rise in pollution levels in major cities worldwide, we are likely going to face even greater challenges regarding this issue. Clearly, it appears that environmental chemicals are harmful to fetal development and this is a key public health challenge that health professionals are facing worldwide. However, health professionals alone may have a limited ability to develop interventions or find solutions to prevent exposure to toxic chemicals during pregnancy. Instead, more coordinated efforts by all those involved in prenatal care and early pregnancy including broad level interventions by governments might be required to prevent toxic exposure during pregnancy.

Although our findings on the detrimental impact of toxic chemical exposure on pregnancy were in line with the studies conducted around the world, a unique aspect of our result was the significant association between *khat* chewing during the periconceptional period and an increased risk of having congenital malformed infants. Those women who have been chewing *khat* during their periconceptional period were three times more likely to have malformed infants as compared to their counterparts.

A review of the literature indicated that this is a clear and significant public health issue facing many African countries [14, 21, 30]. For example, in line with our findings, Yemeni pregnant women who chewed *khat* were 2.02 times more likely to give malformed infants than women who did not chew *khat* [31]. Similarly, it is also in agreement with other reports that showed substance use during pregnancy was significantly associated with the occurrence of congenital anomalies [32, 33].

In the region where our study was conducted, *khat* chewing was a common practice by women, even during pregnancy, which could have a damaging effect on fetal development [34]. There are also deep-rooted cultural, social and traditional values for this highly popular but damaging activity. We believe that this may be one of the biggest challenges facing public health officials in Ethiopia, but it is the one that is extremely important to pursue. Folic acid supplementation in early pregnancy may help decrease the risks of congenital anomalies [35], and mothers should be counseled to eat foods fortified with folic acid.

### Limitations of the study

Despite its strengths, this study has several limitations that must be acknowledged. Although our study had a diverse set of participants, an unmatched case-control study design may be affected by many confounding factors and the results may be less reliable, and we were also not able to consider some congenital malformations that could be detected later in life. It would be useful to consider such populations in future studies. Furthermore, the diagnosis of CMs relied only on clinical examinations, and cytogenetic and metabolic analysis were not performed as these procedures were not available in the study hospitals. Hence, the study is likely to miss some congenital anomalies. The authors also acknowledge the limitations associated with the timing and duration of environmental exposure of mothers and self-reported data.

## Conclusions

Although our study has not addressed the issue of preventing CM during pregnancy, it has identified some of the modifiable risk factors that place pregnant women at high risk of giving malformed infants. Maternal exposure to pesticides during pregnancy and *khat* chewing during the periconceptional period are two important modifiable risk factors that were significantly associated with congenital anomalies. Hence, due attention should be given to public health interventions targeting *khat* chewing and maternal exposure to pesticides during pregnancy.

## Supplementary information

**Additional file 1.** English language version of the consent form and questionnaires which was developed for this study.

## Data Availability

All data generated/analyzed during this study are included in this published article. Besides, part of the row datasets will be available from the corresponding author on a reasonable request.

## Abbreviations

AOR: Adjusted Odds Ratio
CI-: Confidence Interval
CMs: Congenital Malformation
COR: Crude Odds Ratio
SPSS: Statistical Package for the Social Sciences

## Footnotes

1 Khat is a plant and its leaves and stem are chewed by people to elevate mood. It is mostly practiced in East Africa and the Arabian countries.
