# Supplementary material for: Modifiable risk factors of congenital malformations in bale zone hospitals, Southeast Ethiopia: an unmatched case-control study
Source: BMC Pregnancy Childbirth. 2020 Feb 27;20:129. doi: 10.1186/s12884-020-2827-0 (PMC7045613; doi:10.1186/s12884-020-2827-0)
Supplement: Supplementary file 1 — Additional file 1. English language version of the consent form and questionnaires which was developed for this study. [file 12884_2020_2827_MOESM1_ESM.docx]

# **English version of the consent form and questionnaires.**

Dear Madam -----------------------------

I am ------------ and currently working in this hospital. The aim of this study is to generate evidence on ‘modifiable risk factors of congenital malformations in Bale zone Hospitals”. Findings from this study will contribute to the much needed evidence on modifiable risk factors of congenital malformations. It is anticipated that this research will contribute to bridging the information gap about this problem in our country as there have not been studies done addressing this particular important issue.

Thus this interview is prepared for this purpose to get appropriate information on the topic. The information that will be obtained using this interview will be used only for research purpose. Confidentiality and anonymity is fully assured, as your name is not required and only the research team will have access to the results. It will not affect you in anyway, should you not take part in this study? **If yes continue to sign consent form** If No, stop here

I have been informed that the purpose of this study is assessing modifiable risk factors of congenital malformations in Bale Zone hospitals, South East Ethiopia. I understood that participation in this study is entirely voluntarily. I have been told that my answer to the question will not be given to anyone else and no reports of this study identify me in any way. I understood that participation in this study does not involve risks. I understood that Alemayehu Gonie is a contact person if I have question about the study or about my right as a study participant.

Respondent’s Signature ___________Date___________Start interview.

Supervisor’s name ________________ signature _________

**Address of principal investigator:** **Tell**: 0912379531 **e-mail:** [alemayehugonie19@gmail.com](mailto:alemayehugonie19@gmail.com)

| **Code ፡ 1= case 2= control** | **Hospital code፡ 1. Goba 2. Robe 3. Ginir 4. Dolomena** |
| --- | --- |
| **Note: for cases, please put the diagnosis ____________________________________________** | |

| Sr. | **Part I: Demographic Characteristics** | **Options** | | **Remark** |
| --- | --- | --- | --- | --- |
| 1.1 | Age of respondents (in years) | ------- | |  |
| 1.2 | Religion | 1=Muslim 2=orthodox 3= protestant 4= others | |  |
| 1.3 | Marital status | 1=single 2= married | |  |
| 1.4 | Educational level | 1= no formal schooling 2= primary school  3=high school 4= college education | |  |
| 1.5 | Occupation of women | 1= Housewife 2= Farmer 3= Merchant  4=Employee (government/private employee) | |  |
| 1.6 | Monthly income in cash | ------------------ | |  |
| 1.7 | Residence | 1=Urban 2= Rural (Wereda Name-------- | |  |
| 2. | **Part II: Obstetrics Characteristics** | | |  |
| 2.1 | Gravida (in number) | | ------------ |  |
| 2.2 | Parity (in number) | | ------------ |  |
| 2.3 | Current birth Gestational age in weeks | | ------------ |  |
| 2.4 | Type of delivery | | 1=Single 2=Multiple |  |
| 2.5 | Fetal Sex | | 1=Female2=Male 3=Undefined |  |
| 2.6 | Abortion history/pregnancy loss before 28 wks | | 1= yes 2= no 3=no pregnancy |  |
| 2.7 | Did you have previous pregnancy neonatal death *(note: if she had had pregnancy)* | | 1= yes 2= no 3=no delivery |  |
| 2.8 | Did you have family history of anomalies? | | 1= yes 2= no 3=don’t know |  |
| 2.9 | How many ANC follow up did you have? | | 1. 2. 3. 4. =>5. |  |
| 2.10 | Did you use contraceptives before this pregnancy? | | 1= yes 2= no |  |
| 3. | **Part III: Maternal Medical Histories** | |  |  |
| 3.1 | Did you have diagnosed with hyperthyroid disorder? | | 1= yes 2=no 3=don’t know |  |
| 3.2 | Did you have history of pregnancy induced hypertension*?* | | 1= yes 2=no 3=don’t know |  |
| 3.3 | Did you have history of diabetes? | | 1= yes 2=no 3=don’t know |  |
| 3.4 | Did you have diagnosed with Anaemia? | | 1= yes 2=no 3=don’t know |  |
| 4 | **Medication use** | |  |  |
| 4.1 | Did you use **Anticonvulsants/sedatives** during the current pregnancy? | | 1= yes 2=no |  |
| 4.2 | Did you use **Antibiotics** during the current pregnancy? | | 1= yes 2=no |  |
| 4.3 | Did you use **Analgesics/antipyretics** during the current pregnancy? | | 1= yes 2=no |  |
| 4.4 | Use of other drugs () during the periconceptional period? | | 1= yes 2=no |  |
| 5 | **Folic acid use** | |  |  |
| 5.1 | Did you **Use folic acid** around the periconceptional period (between 1 months before and after last menstrual period) | | 1. Yes 2. No |  |
| 5.2 | Did you use **Iron** during the current pregnancy? | | 1. Yes 2. No |  |
| 6 | **Toxic or Environmental Exposures** | |  |  |
| 6.1 | Did you exposed to **Pesticides** during the current pregnancy? | | 1= yes 2=no |  |
| 6.2 | Did you smoke **Cigarette** during the current pregnancy? | | 1= yes 2=no |  |
| 6.3 | Did consume/drink **alcohol** during the current pregnancy? | | 1= yes 2=no |  |
| 6.4 | Did you ever chewing **Khat** during the periconceptional period? | | 1= yes 2=no |  |
| 6.5 | Did you exposed to **Organic solvents (chemicals used in industries፣ industrial installations and/or have contact with contaminated water, soil, air, or food)** during the current pregnancy? | | 1= yes 2=no |  |
| 6.6 | Did you exposed to **Heavy metals (mercury, lead, arsenic, aluminum)** during the current pregnancy?  **(***Exposed to broken fluorescent light, glass thermometers contain mercury, some cosmetics contain lead, particularly lipsticks, baking powders, processed cheeses, )* | | 1= yes 2=no |  |
| 6.7 | Did you undergone X-ray**/radiation therapy** during the current pregnancy? | | 1= yes 2=no |  |
| 6.8 | Did you use separate Cooking kitchen | | 1= yes 2=no |  |
| 6.9 | Is there any ventilation during heating/cooking? | | 1= yes 2=no |  |
| 6.10 | Did you Use of **coal stove** for heating | | 1= yes 2=no |  |
| 7 | **Dietary habits** | |  |  |
| 7.1 | How match **Meat** did you consume (including sea food) during the current pregnancy? | | 1= <1 meal per week  2=1–3 meals per week  3=≥4 meals per week  4= don’t know |  |
| 7.2 | How match **Egg/milk** did you consume during the current pregnancy? | | 1= <1 meal per week  2=1–3 meals per week  3=≥4 meals per week  4= don’t know |  |
| 7.3 | How match **Fresh vegetable** did you consume during the current pregnancy? | | 1= <1 meal per week  2=1–3 meals per week  3=≥4 meals per week  4= don’t know |  |
| 7.4 | How match **Fresh fruit** did you consume during the current pregnancy? | | 1= <1 meal per week  2=1–3 meals per week  3=≥4 meals per week  4= don’t know |  |
| 7.5 | How match **Legume** did you consume during the current pregnancy? | | 1= <1 meal per week  2=1–3 meals per week  3=≥4 meals per week  4= don’t know |  |
| Thank you for your participation!!! | | | |  |
